# Refined Temporal-to-Frontal Horn Shunt for Treatment of Trapped Temporal Horn After Surgery of Peri- or Intraventricular Tumor: A Case Series Study

**DOI:** 10.3389/fonc.2021.781396

**Published:** 2021-11-25

**Authors:** Xiaohui Ren, Yong Cui, Chuanwei Yang, Zhongli Jiang, Song Lin, Zhiqin Lin

**Affiliations:** ^1^ Department of Neurosurgery, Beijing Tiantan Hospital, Capital Medical University, Beijing, China; ^2^ Beijing Neurosurgical Institute, Capital Medical University, Beijing, China; ^3^ Department of Neurosurgery, Longyan First Affiliated Hospital of Fujian Medical University, Fujian, China

**Keywords:** hydrocephalus, trapped temporal horn, surgical treatment, temporal to-frontal horn shunt, outcome, intraventricular tumor

## Abstract

**Background:**

Trapped temporal horn (TTH) is a localized hydrocephalus that can be treated with cerebrospinal fluid diversion. Refined temporal-to-frontal horn shunt (RTFHS) through the parieto-occipital approach is rarely reported in the literature and its effectiveness remains unclear. The aim of the present study is to investigate the efficacy and outcome of RTFHS for treatment of TTH.

**Materials and Methods:**

We consecutively enrolled 10 patients who underwent RTFHS for TTH after surgical resection of peri- or intraventricular tumors from February 2018 to March 2021. Clinical, radiological, and follow-up data were collected and analyzed. The most common underlying pathology was meningioma (n=4), followed by central neurocytoma (n=3), thalamic glioblastoma (n=2), and anaplastic ependymoma (n=1).

**Results:**

The mean Karnofsky performance scale (KPS) score and TTH volume at onset were 54.0 ± 15.1 (range 40-80) and 71.3 ± 33.2cm^3^ (range 31.7-118.6cm^3^), respectively. All patients (10/10, 100.0%) presented with periventricular brain edema (PVBE), while midline shift was observed in 9 patients (9/10, 90.0%). RTFHSs were implanted using valveless shunting catheters. No patients developed acute intracranial hemorrhage or new neurological deficit postoperatively. During the follow-up of 17.2 ± 13.7 months (range 3-39 months), all patients showed clinical and radiological improvement. The mean KPS score at the last follow-up was significantly increased to 88.0 ± 10.3 (range 70-100, p<0.0001). RTFHS resulted in significant complete remission in PVBE and midline shift in 8 (80.0%, p=0.0007) and 9 (100.0%, p=0.0001) patients, respectively. As the postoperative follow-up duration prolonged, the mean TTH volume decreased in a consistent, linear trend (p<0.0001). At last follow-up, the mean TTH volume was significantly reduced to 15.4 ± 11.5 cm^3^ (range 5.6-44.1 cm^3^, p=0.0003), resulting in a mean relative reduction of 77.2 ± 13.1% compared with the volume of TTH at onset. Over drainage was not observed during the follow-up. No patient suffered from proximal or distal shunt obstruction or shunt related infection, and the revision rate was 0%.

**Conclusion:**

RTFHS seems to be safe and effective for the treatment of TTH with favorable outcomes. Advantages of this technique could be technically less complex and invasive, cost-effective, avoidance of various intraperitoneal complications, and maintaining a near-physiological CSF pathway.

## Introduction

Trapped temporal horn (TTH) can be a complication after surgery of lesion within or adjacent to the lateral ventricular trigone (1–3). The obstruction of trigone outlet with continuous CSF production in a relatively closed fluid space lead to dilatation of the temporal horn (4).

As a localized hydrocephalus, TTH can be managed with CSF shunting to extracranial compartments, most commonly the peritoneal cavity (5). However, the conventional ventriculo-peritoneal shunt (VPS) is not ideal with high revision rates on long-term follow-up (6–12). Temporal-to-frontal horn shunt (TFHS) was firstly reported by Hervey-Jumper et al. in 2010 as a feasible strategy for TTH (2). Shunting the CSF from the temporal horn to the frontal horn could mimic the physiological conditions with the added advantages of avoiding intraperitoneal complications and spread of malignant tumor cells. Nevertheless, the described entry point into the temporal horn through the squamous temporal bone carries the risk of injuring the sylvian veins and the middle cerebral artery and its branches (2, 13). Frazier’s point, located 6 cm superior to the inion and 3 cm lateral from the midline, is a safe and established anatomic landmark for placing a frontal catheter through parieto-occipital approach in a VPS. Therefore, penetrating the temporal horn through the Frazier’s point could theoretically afford a safer trajectory than through the squamous temporal bone. However, few reports involving this technique are available in the literature and its effectiveness remains unclear.

The freehand technique for frontal catheter insertion is based on fixed anatomical landmarks and does not take individual variation, such as midline shift and distorted ventricle, into consideration. Neuro-navigation has been applied and improved the accuracy of catheter insertion. However, this technique requires more time and resources (14). A patient-tailored approach based on the use of augmented-reality techniques can address this shortcoming. The Sina neurosurgical assist (Sina), a precise and simple Android application in smartphone, has been reported and utilized for intraoperative neurosurgical planning aid (15).

The aim of the present study is to describe the technique of refined temporal-to-frontal horn shunt (RTFHS) through the parieto-occipital approach with assistance of Sina application and report the preliminary experiences on the efficacy and outcome of RTFHS for the treatment of TTH.

## Methods

### Patient Population and Data Collection

We retrospectively analyzed the clinical records of 10 consecutive patients who underwent RTFHS for TTH after surgery of peri- or intraventricular tumors between February 2018 and March 2021 at our institution. Medical records and radiological findings were reviewed. The underlying pathology included meningioma in 4 cases, central neurocytoma in 3 cases, thalamic glioblastoma in 2 cases, and anaplastic ependymoma in 1 case. The neurological status was evaluated with Karnofsky performance scale (KPS). CT and/or MRI were employed for diagnosis of TTH preoperatively and to monitor TTH evolution in the following days. The TTH volume index was calculated according to the formula for the volume of a spheroid: 4/3 × π × (length/2) × (width/2) × (height/2). Reduction of the TTH volume, and periventricular brain edema (PVBE) were defined and calculated as reported in the previous study (5). This study was approved by the Research Ethics Board of Beijing Tiantan Hospital, Capital Medical University. Written informed consent was obtained from all patients.

### Surgical Procedure

**Figure 1:**
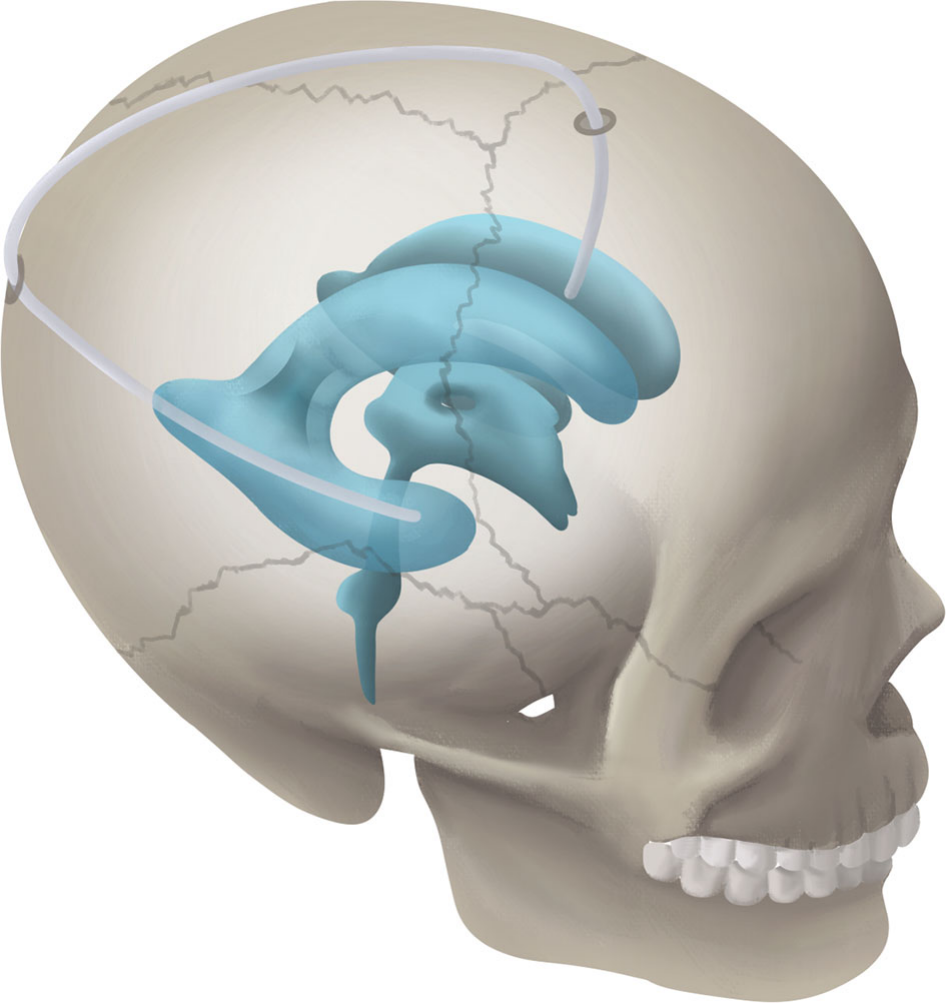
Schematic diagram showing the RTFHS technique, shunting CSF from temporal horn to frontal horn.

**Figure 2:**
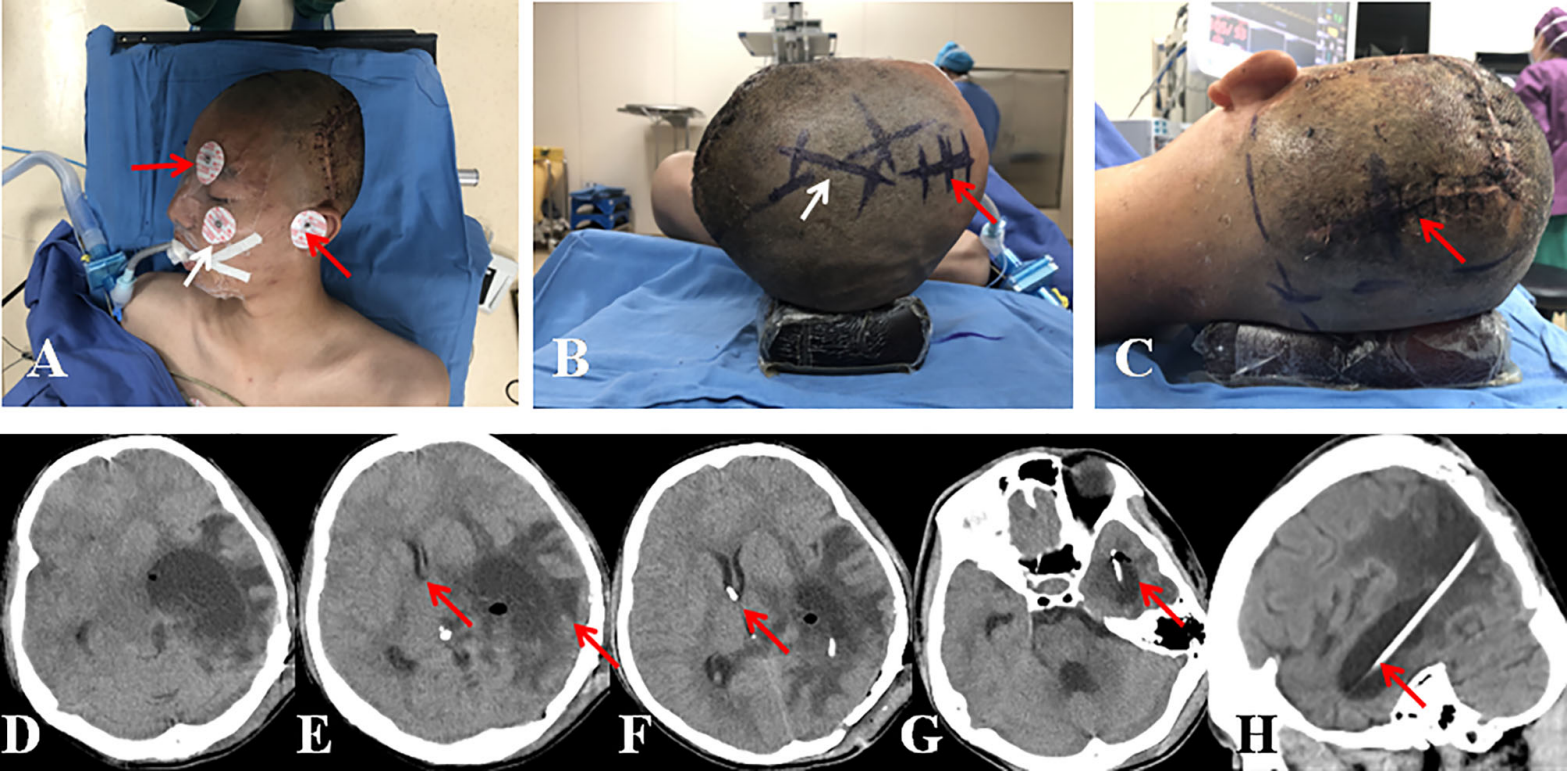
**(A)** Patient position in superior view (red arrow, landmarks of medial canthus and tragus; white arrow, landmark of zygomatic process). **(B)** Patient position in rostral view (red arrow, skin incision for frontal burr hole; white arrow, skin incision for subcutaneous connection of the temporal and frontal catheters). **(C)** Patient position in left view (red arrow, skin incision for occipito-parietal burr hole). **(D)** CT scan showed severe dilation of the left temporal horn with compression of the brainstem. **(E)** CT scan showed prominent midline shift, PVBE, and displaced frontal horn (red arrows). **(F, G)** CT scan showed the suggested localization of catheter tips in the frontal and temporal horns (red arrows). **(H)**: CT scan showed the trajectory of temporal catheter (red arrow).

Schematic diagram of RTFHS technique is illustrated in **Figure 1**. The patient position, skin incision, and catheter localization are shown in **Figure 2**. The patient is placed in the supine position with head turned opposite the side of the affected temporal horn. The head should be rotated 60° to 70°, as is illustrated in **Figure 2A** (superior view), 2B (rostral view), and 2C (left view). The key anatomic landmarks for placing a frontal and temporal catheter include the medial canthus, the tragus, and the zygomatic process. Two burr holes are created. The proximal catheter (Medtronic, Inc., Minneapolis, Minnesota) is placed in the temporal horn from a parieto-occipital approach (Frazier’s point, located 3 cm lateral from the midline and 6 cm superior to the inion) and the target point is the zygomatic process of the ipsilateral face (white arrow in **Figure 2A**). Then, the catheter passes the planned trajectory with a depth of approximately 10.5 cm as measured from the level of the dura. The catheter tip rests near the front wall of temporal horn, away from the rich choroid plexus of trigone. The distal catheter is inserted into the frontal horn through an usual frontal approach (Kocher’s point, located 2.5 cm anterior to the coronal suture and 2.5 cm lateral from the midline). The trajectory is toward the medial canthus of the ipsilateral eye on the coronal plane and a point 1 cm anterior to the tragus on the sagittal plane. The catheter advances for a depth of 5 cm and lies in front of the foramen of Monro. A middle skin incision (white arrow in **Figure 2B**) was made for subcutaneous connection of the proximal and distal catheters by a straight connector without a programmable anti-siphon valve. The ideal catheter tip localization in the frontal and temporal horn were showed in **Figures 2F, G**, respectively. The orientation of catheter in the temporal horn was shown in **Figure 2H**.

In some cases, the TTH may presents as giant mass (**Figure 2D**), leading to severe PVBE and midline shift (**Figure 2E**). The displaced, distorted, and small frontal horn makes it difficult to place a frontal catheter through a freehand technique. Then, the Sina neurosurgical assist (Sina) application was introduced to provide guidance and continuous monitoring during insertion of the frontal catheter. The Sina application was used according to the method described by Eftekhar B (15). Briefly, the appropriate axial and coronary CT or MRI slices are selected at the level of the foramen of Monro. Photographs of these images are taken in portrait mode. With the application assist, the orientation of the puncture was marked with electrode stickers (**Figure 2A**). The unscrubbed assistant overlaps the coronal radiological image onto the real-time view of the patient’s head. The device is held by an assistant who aligns the images and provides information about the relative position of the target and frontal catheter to the surgeon.

### Statistical Analysis

The demographics and clinical characteristics were described in terms of means ( ± SD) and frequencies. Fisher’s exact test and Wilcoxon matched-pairs signed ranks test was used to assess the differences of clinical and radiological data between TTH at onset and at last follow-up. The statistical software SPSS 13.0 (SPSS for Windows, version 13.0 [SPSS Inc., Chicago, Illinois]) was used. Probability values were reported as 2 sided, with statistical significance defined as *P*<0.05.

## Results

### Baseline Characteristics

There were 4 male and 6 female with a mean age of 40.0 ± 22.1 years. The mean time interval from tumor resection to TTH onset was 5.2 ± 6.6 months (range 8 days-19 months). Symptoms of intracranial hypertension was the most common presentation (60.0%). The mean KPS score at onset was 54.0 ± 15.1 (range 40-80). Eight TTHs (80.0%) were located in the left hemisphere and 2 (20.0%) in the right. Moderate and severe PVBE were presented in 3 (30.0%) and 7 (70.0%) patients, respectively. Midline shift was observed in 9 cases (90.0%), and the mean distance of midline shift was 6.2 ± 4.1 mm (range 0-11mm). The mean TTH volume at onset was 71.3 ± 33.2cm^3^ (range 31.7-118.6cm^3^). The clinical data are summarized in **Table 1**.

**Table 1:** Baseline characteristics of patients with trapped temporal horn who were treated with refined temporal-to-frontal horn shunt^a^.

Case No.	Sex/age(years)	Underlying pathology	Presentation	PVBE	FU (months)	Midline shift (mm)	TTH volume at onset (cm^3^)	Mean volume reduction at 2 weeks (%)	TTH volume at last FU (cm^3^)	Mean volume reduction at last FU (%)	KPS before shunt/at last FU
1	M/30	Central neurocytoma	ICH, seizure	severe	39	6	114.6	60.4	14.2	87.6	50/90
2	M/17	Central neurocytoma	Blurred vision	moderate	38	3	68.6	18	9.6	86	70/100
3	F/25	Central neurocytoma	Memory disturbance, right limb weakness	severe	24	8	91.2	72.2	14.5	84.1	60/100
4	F/64	Thalamic glioblastoma	ICH, herniation, aphasia	severe	20	10	84.8	35.2	25	70.5	50/70
5	F/38	Meningioma	Vertigo, memory disturbance	moderate	22	0	34	22.2	9.8	71	70/90
6	M/17	Anaplastic ependymoma	ICH	severe	11	10	118.6	73	10.3	91.4	40/90
7	M/71	Thalamic glioblastoma	ICH, herniation	severe	5	10	40.4	91.9	5.6	86.2	40/70
8	F/16	Meningioma	Memory disturbance	moderate	7	2	89.2	18	44.1	50.6	80/90
9	F/57	Meningioma	ICH, herniation, aphasia, right limb weakness	severe	3	11	31.7	74.8	5.7	82	40/90
10	F/65	Meningioma	ICH, memory disturbance; mental symptoms	severe	3	2	39.9	48	15	62.4	40/90

a TTH, trapped temporal horn; PVBE, periventricular brain edema; FU, follow-up; ICH, intracranial hypertension; KPS, Karnofsky Performance Scale.

### Surgical Complications and Outcomes

**Figure 3:**
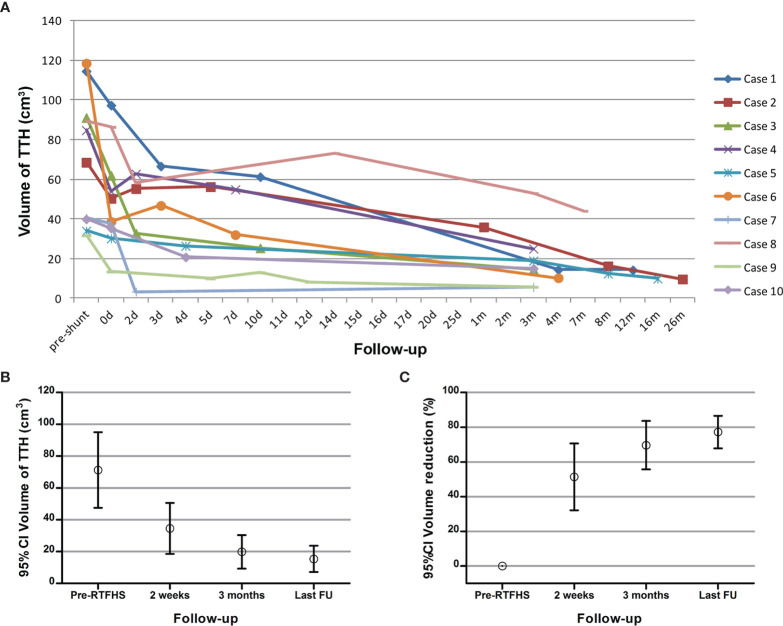
**(A)** The volume curve of the TTH from each patient during the follow-up. **(B, C)** The mean TTH volume and the mean reduction of TTH volume before shunt, at 2 weeks, at 3 months, and at last follow-up.

No patients developed acute intracranial hemorrhage or new neurological deficit postoperatively. During the follow-up of 17.2 ± 13.7 months (range 3-39 months), all patients showed clinical and radiological improvement. The mean KPS score at the last follow-up was 88.0 ± 10.3 (range 70-100). All patients demonstrated immediate and further radiographic resolution after RTFHS (**Figure 3A**). The mean TTH volume at 2 weeks, 3 months, and the last follow-up was 34.5 ± 22.4 cm^3^ (range 3.3-73.2 cm^3^), 19.8 ± 14.8cm^3^ (range 5.6-53.0 cm^3^), and 15.4 ± 11.5 cm^3^ (range 5.6-44.1 cm^3^), respectively (**Figure 3B**). The mean volume reduction at 2 weeks, 3 months, and the last follow-up was 51.4 ± 26.9% (range 18.0-91.9%), 69.6 ± 19.6% (range 40.6-91.4%), and 77.2 ± 13.1% (range 50.6-91.3%), respectively (**Figure 3C**). PVBE complete resolution and return of the midline to a normal position were observed in 8 cases (80.0%, 8/10) and 9 cases (100.0%, 9/9), respectively. Over drainage was not observed during the follow-up. The revision rate was 0%; in no case was a proximal or distal shunt obstruction or shunt related infection encountered.

### Differences Between the Preoperative and Postoperative Data

The mean KPS score was significantly increased after RTFHS (54.0 ± 15.1 at onset *vs* 88.0 ± 10.3 at the last follow-up, p=0.0057). Radiologically, RTFHS resulted in a significant complete remission in PVBE (p=0.0007) and midline shift (p=0.0001). In addition, the mean TTH volume at last follow-up was significantly reduced (71.3 ± 33.2cm^3^ at onset *vs* 15.4 ± 11.5 cm^3^ at last follow-up, p=0.002). As the postoperative follow-up duration prolonged, the mean TTH volume decreased in a consistent, linear trend (**Figure 3B**, p<0.0001).

### Illustrative Cases

#### Case 6

**Figure 4:**
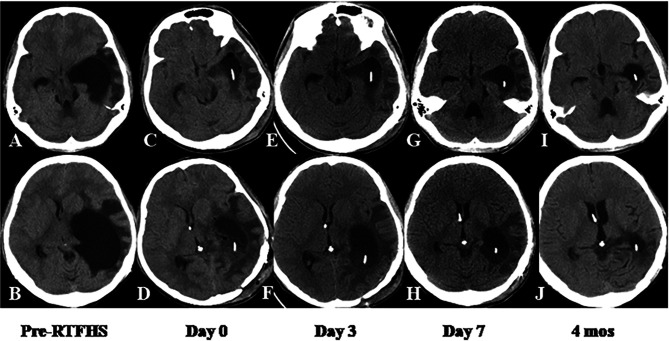
Case 6. CT scan showed immediate and further reduction of TTH volume at the level of midbrain (upper panel) and Foremen of Monro (lower panel). The volume and morphometric changes of TTH were shown before RTFHS **(A, B)**, at Day 0 **(C, D)**, Day 3 **(E, F)**, Day 7 **(G, H)**, and 4 months **(I, J)**.

This 17-year-old male underwent craniotomy for resection of anaplastic ependymoma in the left lateral ventricular trigone. Eight days following surgery, he developed symptoms of intracranial hypertension. A CT scan revealed a giant TTH with prominent PVBE and mildline shift (**Figures 4A, B**). RTFHS was performed. Postoperatively, the patient’s symptoms resolved. A series of CT scan showed gradual resolution of PVBE and immediate and further reduction of TTH volume at the level of midbrain (upper panel) and Foremen of Monro (lower panel) (**Figures 4C–J**). The volume reduction at Days 3, Day 7, and 4 months were 60.8%, 73.0%, and 91.3%, respectively.

## Discussion

### The Current Difficulties in Treating the TTH

TTH is a rare entity and only a few studies with a limited number of patients have studied the surgical intervention for it. Surgical treatment options are diversely described in the literature, ranging from microsurgery *via* craniotomy to CSF diversion to neuroendoscopic techniques (2, 4, 16–20). To date, the optimal surgical modality is yet to be definitively determined. Some patients might even require multiple operations until the situation is well controlled. Microsurgical fenestration through craniotomy enables to open the scarred trigone and remove the ventricular septation (4, 5). Furthermore, simultaneous choroid plexectomy reduced the production of CSF from the temporal horn. Nevertheless, this technique is traumatic and carry the risk of injury to the surrounding critical structures. Endoscopic fenestration of the choroidal fissure has been described; however, it may sometimes limited by technically challenging, surgeon experience, and unknown long-term patency of the stoma (18, 19). Besides, this technique should takes the individual anatomical variation into account (19). Although VPS remains to be the mainstay of CSF diversion for treatment of TTH, there are risks specific to VPS, including intraperitoneal complications, malfunction, infection, and dependence in the long-term follow-up (1, 21).

### The Novelty, Safety, and Feasibility of RTFHS

The efficacy of TFHS have been firstly illustrated by Hervey-Jumper et al. in 2010 as a report of 3 cases (2). They advocated that this technique could be considered in patients with neurological symptoms resulting from TTH secondary to peri- or intraventricular malignant tumors in which seeding of distant sites by CSF diversion is a concern. With regard to the entry point into the enlarged temporal horn, a bur hole on the squamous temporal bone was suggested in this study (2). However, the described perpendicular or lateral approach harbored the risk of damaging the sylvian veins and the middle cerebral artery and its branches (2). Therefore, the safety and feasibility of TFHS for a greater number of patients are still unclear. In addition, the overshooting catheter might result in injury to the midbrain and even some critical structures in the surrounding cisternal system, such as basilar artery, oculomotor nerve, posterior communicating artery, and anterior choroidal artery (13). Furthermore, it cannot ensure the catheter permanently long enough within the temporal horn, especially for a decompressed TTH following shunt. Frazier’s point has been established as a common and safe anatomic landmark for placing a frontal catheter through parieto-occipital approach in VPS. Then, we proposed the RTFHS by penetrating the temporal horn through the Frazier’s point. The occipito-parietal approach paved a safe trajectory and enabled the catheter away from the sylvian vessels, midbrain, and some other critical structures. Moreover, a longer shunt path in the temporal horn and keeping the catheter tip away from the rich trigonal choroid plexus prevented catheter displacement and obstruction. No patients experienced acute intracranial hemorrhage or new neurological deficit associated with RTFHS in this case series. These results support the safety of RTFHS.

Another critical stage of RTFHS procedure is the insertion of the distal catheter. In our series, the mean TTH volume at onset was 71.3 ± 33.2cm^3^ and more than half of the cases presented with severe PVBE. The giant TTH with brain edema inevitably led to midline shift and displaced ventricle. As such, placing a frontal catheter through a freehand technique can be challenging. Although stereotactic navigation and image guidance ensure a more accurate placement of the ventricular catheter, they impose a longer duration of plan and surgery, require more operating room resources utilization, and increase the patients’ health care costs. Then, Sina application, a precise and available software in smartphone, was used as a simple intraoperative neurosurgical planning aid in placement of catheter. We did not experience difficulty in placing a frontal catheter in cases with severe midline shift or distorted ventricle. Sina application-assisted Kocher’s point puncture is used to perfectly locate the catheter, taking the place of neuro-navigation. This application not only offers a simple, realistic, and available manner in the placement of shunt, but also demonstrates high degrees of achieved accuracy. In our series, midline shift was observed in 9 cases (90.0%), 4 of which demonstrated a severe degree. Nevertheless, successful penetration of the frontal horn was achieved in all cases and the postoperative imaging confirmed the distal end in position.

The present study included a much larger number of cases treated with TFHS than prior studies. Clinical improvement was achieved immediately after shunt insertion. TTH volume, PVBE, and midline shift were substantially improved at the last follow-up. No patients suffered from proximal or distal shunt obstruction or shunt related infection during the follow-up. Our experience demonstrates that the refined and modified technique can be a safe, effective, and durable CSF diversionary procedure. Further prospective multicenter study with more cases and extended follow-up will be necessary to validate this technical approach.

### Advantages of RTFHS Compared With Conventional VPS

The RTFHS has several advantages over the conventional VPS. First, the shunt system in RTFHS is shorter in length and confined to the skull, which makes the operation technically less complex and invasive and minimizes the risk of mechanical failure and infection. Second, siphoning phenomenon and overdrainage always occur in patients with VPS and have not been eliminated despite with the use of antisiphoning devices (6–9). RTFHS decreases the pressure gradient, avoids shunt siphoning, and prevents overdrainage, maintaining a natural and near-physiological CSF pathway. At the last follow-up, no cases presented with overdrainage in our series. Moreover, without having to use antisiphoning device and programmable valve, RTFHS can also significantly decreases the cost of implant. Third, the peritoneum is the cause for VPS revision in many cases on the long-term follow-up. RTFHS obviates distal catheter occlusion and various intraperitoneal complications. In addition, there are high risks for adhesions and surgical bowel perforation in patients with multiple previous abdominal surgeries or with a previous abdominal shunt infection. RTFHS can be an effective alternative in treating these patients. Finally, in cases of TTH caused by primary peri- or intraventricular malignant tumors, RTFHS prevents spreading malignant tumor cells to distant sites.

El-Shafei et al. (22) pioneered the technique of retrograde ventriculosinus shunt for treatment of hydrocephalus by shunting the CSF to the superior sagittal sinus against the direction of blood flow. A system review by Toma et al. (23) with a total of 265 patients treated with ventriculosinus shunt demonstrated that this technique is safe and feasible, and do not increase the risk of sinus thrombosis, air embolism, uncontrollable intraoperative bleeding, or shunt-associated nephritis. There are several similarities and advantages between the RTFHS and retrograde ventriculosinus shunt since both of them divert CSF back to the intracranial compartment. In our opinion, the temporal horn to sagittal sinus shunt, which is developed and evolved from these two techniques, might be a feasible alternative for treatment of TTH sometimes when a RTFHS is not available. To date, the temporal horn to sagittal sinus shunt has not been reported in the literature. This procedure would be a choice and attempt in selected patients in our future practice.

### Limitations

This was a retrospective review of a rare series of TTH cases that was treated by RTFHS. Although the morphometric changes including TTH volume, PVBE, and midline shift were evaluated, there were some important CSF hydrodynamic parameters that were not investigated due to the retrospective nature of the study. These hydrodynamic parameters can help to gain more insight into the pathophysiology of non-communicating hydrocephalus patients. In recent studies, 3D fluid-structure interaction modeling was utilized to examine the correlation between CSF hydrodynamic changes and non-communicating hydrocephalus patients’ clinical symptoms before and after shunting (24, 25). The ventricular system volume and maximum CSF pressure were found to be more effective and accurate than the other parameters in evaluating the patients’ conditions. Future prospective investigation assessing the hydrodynamic parameters changes of the CSF flow during the treatment process of TTH is warranted. In addition, this series included a small and uncontrolled patient group, which provided limited statistical analysis and comparison between different treatment options. A thorough understanding of the RTFHS technique requires a prospective multicenter study with great number of patients in the future.

## Conclusion

RTFHS seems to be safe and effective for the treatment of TTH with favorable outcomes. RTFHS might be a potential alternative to traditional VPS for patient with TTH. Advantages of this technique could be technically less complex and invasive, cost-effective, avoidance of various intraperitoneal complications, and maintaining a natural and near-physiological CSF pathway. Multicenter prospective study with a great number of patients is necessary to validate the potential benefits of this technique so that it can be widely recommended.

## Data Availability Statement

The original contributions presented in the study are included in the article/supplementary material. Further inquiries can be directed to the corresponding authors.

## Ethics Statement

The studies involving human participants were reviewed and approved by Ethics Committee of Beijing Tiantan Hospital. Written informed consent to participate in this study was provided by the participants’ legal guardian/next of kin. Written informed consent was obtained from the individual(s), and minor(s)’ legal guardian/next of kin, for the publication of any potentially identifiable images or data included in this article.

## Author Contributions

XR, SL, and ZL contributed to conception and design of the study. XR organized the database. XR and ZL performed the statistical analysis. XR and ZL drafted the manuscript. XR and ZL wrote sections of the manuscript. All authors contributed to manuscript revision, read, and approved the submitted version.

## Conflict of Interest

The authors declare that the research was conducted in the absence of any commercial or financial relationships that could be construed as a potential conflict of interest.

## Publisher’s Note

All claims expressed in this article are solely those of the authors and do not necessarily represent those of their affiliated organizations, or those of the publisher, the editors and the reviewers. Any product that may be evaluated in this article, or claim that may be made by its manufacturer, is not guaranteed or endorsed by the publisher.

## Abbreviations

TTH, trapped temporal horn; TFHS, temporal-to-frontal horn shunt; RTFHS, refined temporal-to-frontal horn shunt; VPS, ventriculo-peritoneal shunt; PVBE, periventricular brain edema; KPS, Karnofsky performance scale; CSF, cerebrospinal fluid.
